# MeJA-mediated enhancement of salt-tolerance of *Populus wutunensis* by 5-aminolevulinic acid

**DOI:** 10.1186/s12870-023-04161-7

**Published:** 2023-04-06

**Authors:** Huan Liu, Jingliang Sun, Jixiang Zou, Baisheng Li, Hua Jin

**Affiliations:** College of Environment and Bioresource, Dalian Minzu University, No 18, Liaohexi Road, 116600 Dalian, Liaoning China

**Keywords:** 5-Aminolevulinic acid, MeJA, Salt-tolerance, Endoplasmic reticulum, Flavonoids

## Abstract

**Background:**

5-Aminolevulinic acid (ALA) is a natural and environmentally benign multifunctional plant growth regulator involved in the regulation of plant tolerance to various environmental stresses. This research aimed to explore the molecular mechanisms of salt tolerance in *Populus wutunensis* induced by exogenous ALA using physiological and transcriptomic analyses.

**Results:**

Physiological results showed that 50 mg·L^− 1^ ALA-treatment significantly reduced the malondialdehyde (MDA) content and the relative electrical conductivity (REC) and enhanced antioxidant activities of enzymes such as SOD, POD and CAT in salt-stressed *P. wutunensis* seedlings. Transcriptome analysis identified ALA-induced differentially expressed genes (DEGs) associating with increased salt-tolerance in *P. wutunensis*. GO and KEGG enrichment analyses showed that ALA activated the jasmonic acid signaling and significantly enhanced the protein processing in endoplasmic reticulum and the flavonoid biosynthesis pathways. Results of the hormone-quantification by LC-MS/MS-based assays showed that ALA could increase the accumulation of methyl jasmonate (MeJA) in salt-stressed *P. wutunensis*. Induced contents of soluble proteins and flavonoids by exogenous ALA in salt-treated seedlings were also correlated with the MeJA content.

**Conclusion:**

5-aminolevulinic acid improved the protein-folding efficiency in the endoplasmic reticulum and the flavonoid-accumulation through the MeJA-activated jasmonic acid signaling, thereby increased salt-tolerance in *P. wutunensis*.

**Supplementary Information:**

The online version contains supplementary material available at 10.1186/s12870-023-04161-7.

## Background

5-aminolevulinic acid (ALA) is a non-protein amino acid which is a precursor to the synthesis of tetrahydropyrroles, such as chlorophyll, haemoglobin and vitamin B12 in living organisms and can be synthesized autonomously in all plants, animals and microorganisms [1, 2]. In recent years, ALA has been increasingly used in plant production. It has been shown that ALA can act as a plant growth regulator, promoting the root elongation, the seed germination and the biomass accumulation and increasing crop yield [3–6]. As an endogenous chemical, ALA possesses a wide-range of applications and market-development potentials as it has no toxic effects on human and livestocks, besides it is easily degradable and residue-free in the ecological environment [7].

There are a number of researches indicating that ALA can exert multiple regulatory effects on plant growth, development and responses to environmental stresses [8]. In addition, the external application of ALA can effectively mitigate the adverse effects of abiotic stress, such as drought, high salinity, low temperature, high temperature and heavy metals on plants [9–14]. For example, ALA can alleviate the impairment of chlorophyll synthesis from the conversion of uroporphyrinogen III (UROIII) to protoporphyrin IX (Proto IX) under abiotic stresses [15]. ALA can also enhance the production of chlorophyll and its precursors, the electron transport and the stomatal conductance [16, 17]. Moreover, ALA can suppress the adverse effects of abiotic stress on the photosynthesis [18]. Physiologically, ALA can activate activities of various antioxidant enzymes such as superoxide dismutase (SOD), peroxidase (POD), catalase (CAT) and ascorbate peroxidase (APX), promote efficient AsA-GSH cycling, and reduce malondialdehyde (MDA) and hydrogen peroxide (H_2_O_2_) contents, all of which help to maintain the homeostasis of reactive oxygen species and to alleviate the oxidative stress in plants [19–22]. In the perspective of nitrogen metabolism, ALA is capable of inducing the activities of nitrate reductase (NR), glutamine synthetase (GS), glutamate synthase (GOGAT) and glutamate dehydrogenase (GDH), and suppressing the activity of nitrite reductase (NiR). These enzymatic changes can alleviate the stress-induced cytotoxicity caused by the massive accumulation of nitrate and ammonium [23, 24].

Up to date, most researches on the effects of ALA has focused on the physiological level and little is known on the molecular level. Therefore, it is important to study the molecular mechanism of ALA, the new powerful and widely-used plant growth-regulator. Evidences from the research by Liu et al. (2019a) showed that exogenous ALA could not significantly affect the expression of genes encoding key enzymes for the chlorophyll biosynthesis although it increased chlorophyll content in tomato leaves under the low-temperature [15]. Haianh et al. (2016) also found that the transcriptional levels of most tetrapyrrole-biosynthesis pathway genes remained largely unchanged or slightly decreased after the ALA-treatment in rice [25]. These findings suggest that ALA does not promote photosynthesis and improve plant stress tolerance by acting as a substrate for the chlorophyll synthesis. In addition, ALA can activate the flavonoid biosynthetic pathway and increase the content of antioxidants such as anthocyanins and flavonols in plants, as well as up-regulate the expression levels of anthocyanin transport-related genes, thereby improving fruit quality [26, 27]. Furthermore, the RNA-seq analysis by Wang et al. (2018b) and Niu et al. (2018) found that ALA was also able to attenuate the stress-induced photoinhibition, enhance the PSII function and improve the drought-tolerance through the activation of photosynthesis, photosynthesis-antenna proteins, and carbon fixation in plant [28, 29].

Although ALA can improve the plant abiotic-stress resistance through pathways described above, the detailed molecular mechanisms by which ALA regulates plant growth under stress have not yet been fully elucidated, and particularly the studies on the molecular mechanisms associating with improvement of plant salt-tolerance by ALA have been even less reported.

*Populus wutunensis* cultivated by our research team is widely grown in arid and saline areas [30]. In this study, we conducted RNA-sequencing (RNA-seq) to screen and analyze differentially expressed genes (DEGs) in ALA-treated and salt-stressed *P. wutunensis* seedlings. We explored the signal-transduction and metabolic pathways activated by ALA at an early stage. Our results provided evidence which directly proved that ALA could improve plant stress-tolerance and promote plant growth. Our findings could serve as a theoretical basis for the elucidation of action mechanism of other plant growth regulators and the development of novel plant growth regulators in the future.

## Results

Effect of exogenous ALA on the growth of salt-stressed *P. wutunensis*.

**Fig. 1:**
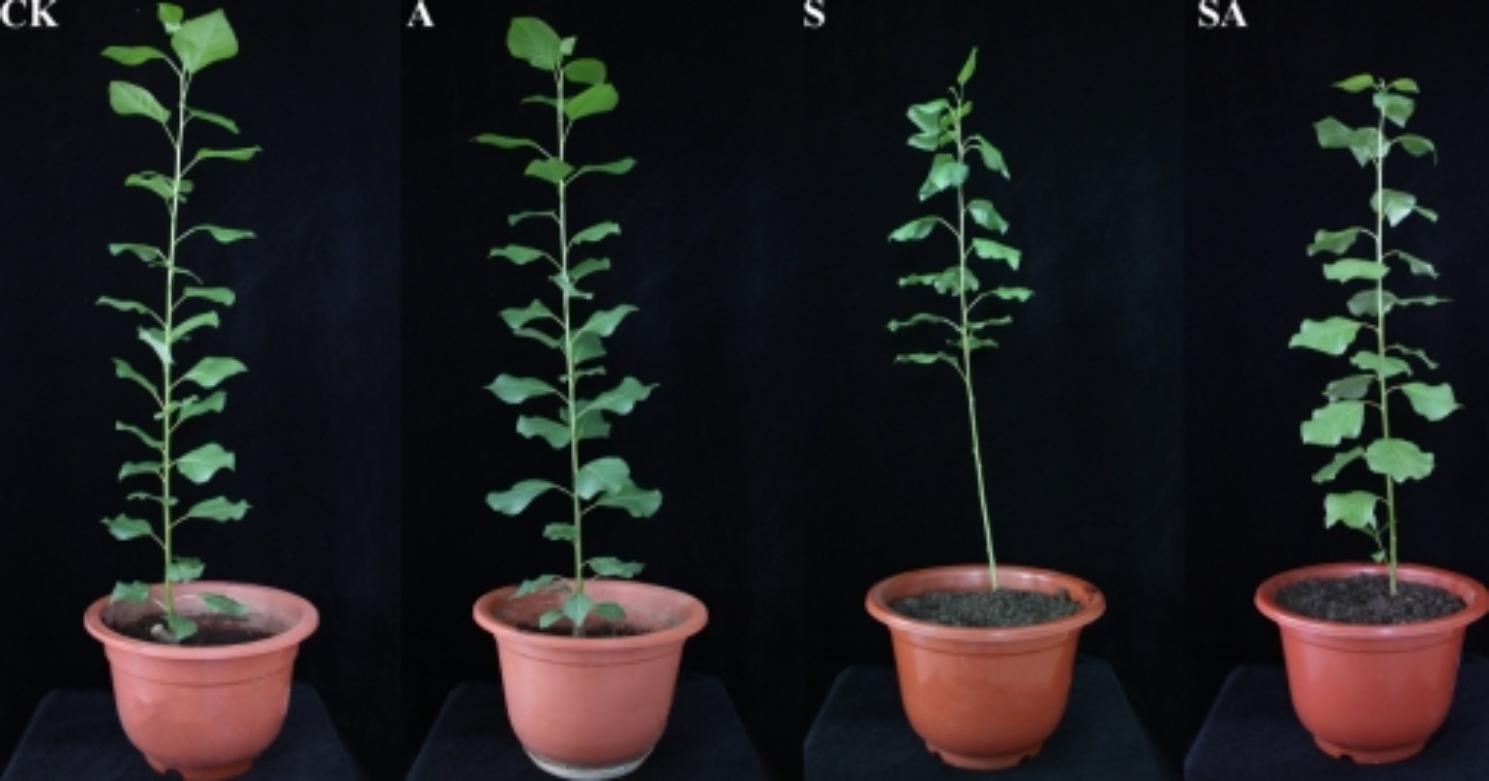
Effect of ALA-treatment on leaf morphology of salt-stressed *P. wutunensis*. Photographs were taken on the 4th day of ALA-treatment.

To examine the effect of exogenous ALA on *P. wutunensis* under the salt stress, the phenotypic and physiological traits of seedlings were analyzed. Under normal conditions, exogenous-spray of ALA had no significant effect on the morphology of *P. wutunensis*. The leaves wilted, curled and even fell off in the group S (salt-stressed) plants. Unlike the group S, although the group SA (salt stress + ALA) also showed wilting and curling, the degree of wilting was lower than that of the group S and the leaf abscission was significantly reduced. These results suggested that the ALA-treatment could positively affect the growth of *P. wutunensis* under the salt stress (Fig. 1).

**Fig. 2:**
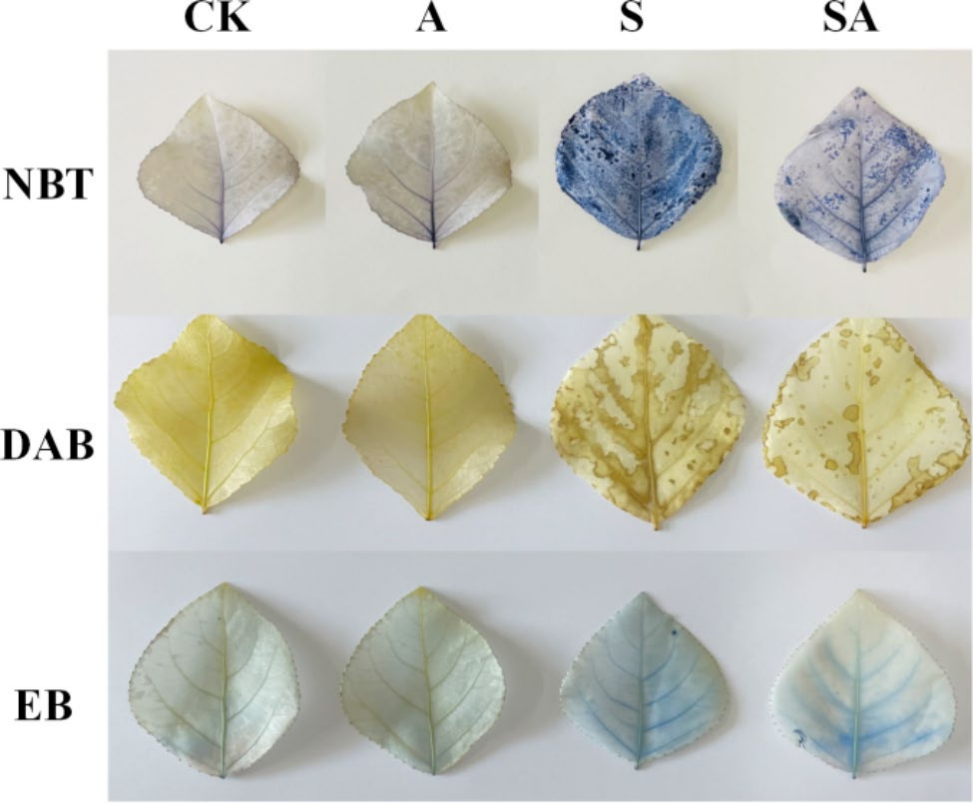
Effect of ALA-treatment on the accumulation of ROS and the extent of cell damage in salt-stressed *P. wutunensis*. Photographs were taken on the 4th day of ALA-treatment.

To investigate the effect of ALA on ROS accumulation and cell damage in leaves, concentrations of superoxide anion and hydrogen peroxide and the degree of cell damage were examined by NBT, DAB and EB, respectively. Compared with CK plants, there was no significant change in the ROS accumulation and cell damage in group A plants. In contrast, plants in the group S exhibited darker staining indicating that the cellular ROS accumulation was significantly higher and cells were more severely damaged than in plants notstressed. The group SA stained darker than the unstressed plants but slightly lighter compared to the group S showing lower ROS accumulation and less cellular damage. This observation suggested that exogenous ALA could reduce the ROS accumulation and cell damage in salt-stressed *P. wutunensis* (Fig. 2).

**Fig. 3:**
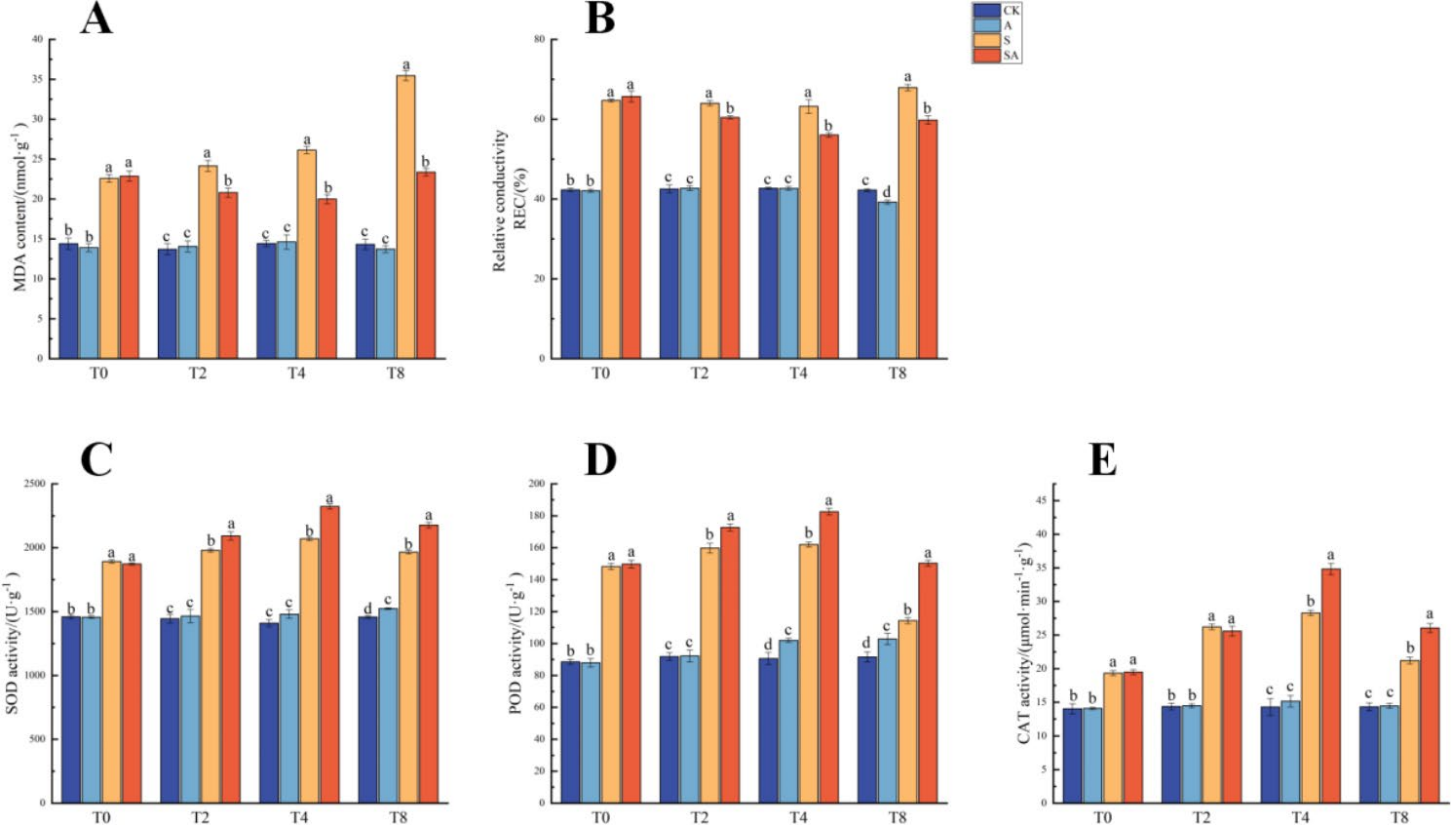
Effects of exogenous ALA on the MDA content, the REC and antioxidant enzyme activities of salt-stressed *P. wutunensis*. (A) MDA content. (B) REC. (C) SOD activity. (D) POD activity. (E) CAT activity. Data were expressed as the mean ± standard error of three independent biological replicates. Different letters indicated significant differences of P < 0.05 according to Duncan test

In addition, the MDA content and relative electrical conductivity (REC) were significantly higher in the group S compared to plants without stress and tended to increase with increasing stress timespans, reaching a maximum at T8 (equivalent to 16 days of salt stress). The activities of SOD, POD and CAT showed an increasing and then decreasing trend with increasing treatment time, reaching a maximum at T4. The decrease in antioxidant enzyme activity at T8 was probably due to prolonged stress. The MDA content and the REC of plants in the group SA showed an increasing trend compared with those in the CK and A groups, but the increase was significantly lower than that in the group S. The activity of antioxidant enzymes in the group SA also tended to increase and then decrease with increasing treatment time, reaching a maximum at T4, but its activity was significantly higher than that of the salt-treated group. The plants in both the CK and A groups were not stressed and there was no significant difference (Fig. 3). These results indicated that exogenous ALA could significantly improve the salt tolerance of *P. wutunensis*, and T4 was the key turning point for exogenous ALA to alleviate salt stress in our study.

Transcriptome analysis of exogenous ALA-treated *P. wutunensis* under salt stress.

To further understand the molecular mechanism of salt-tolerance improvement by exogenous ALA in *P. wutunensis*, transcriptome analysis was performed with leaves from the group SA. Since T4 was the critical turning point for salt-stress alleviation by exogenous ALA, the sampling time points were chosen at 0 (control), 1, 2 and 4 d after ALA treatment. RNAs were extracted from 12 leaf samples (4 treatments × 3 biological replicates) for transcriptome sequencing, respectively.

After removing the low-quality reads, a total of 547,082,188 bp clean reads were obtained (Table S1). The percentages of Q30 and GC were 93.06–93.86% and 44.04–44.42%, respectively, indicating the high quality of the transcriptome sequencing data. The clean read data was used for reassembly. 209,438 transcripts were generated by Trinity software, corresponding to 189,370 unigenes. The average transcript length was 1281 bp and the N50 was 1907 bp (Table S2). All assembled unigenes were compared, annotated and classified against the KEGG, NR, SwissProt, Trembl, KOG, GO and Pfam databases using the BLAST software (Table S3). In addition, 3927 (2270 up-regulations and 1657 down-regulations), 2402 (1217 up-regulations and 1185 down-regulations) and 3486 (1702 up-regulations and 1784 down-regulations) DEGs (|log_2_FoldChange| ≥ 1 and FDR < 0.05) were identified in pairs of T0 vs. T1, T0 vs. T2 and T0 vs. T4 respectively (Fig. S1). It could be concluded that ALA induced significant changes in gene expression levels in *P. wutunensis* under the salt stress.

To further assess the biological function of ALA-induced DEGs under the salt stress, GO and KEGG enrichment analyses were performed on DEGs. The GO categories significantly enriched in DEGs in T0 vs. T1 were “plant-type secondary cell wall biogenesis”, “hemicellulose metabolic process” and “anion homeostasis”. The significantly enriched GO categories in T0 vs. T2 were “galactosidase activity”, “photosystem” and “photosystem II “. The DEGs in T0 vs. T4 were mainly enriched in “endoplasmic reticulum lumen”, “galactosidase activity”, " cell wall modification” and “protein N-linked glycosylation” GO categories (Fig. S2).

**Fig. 4:**
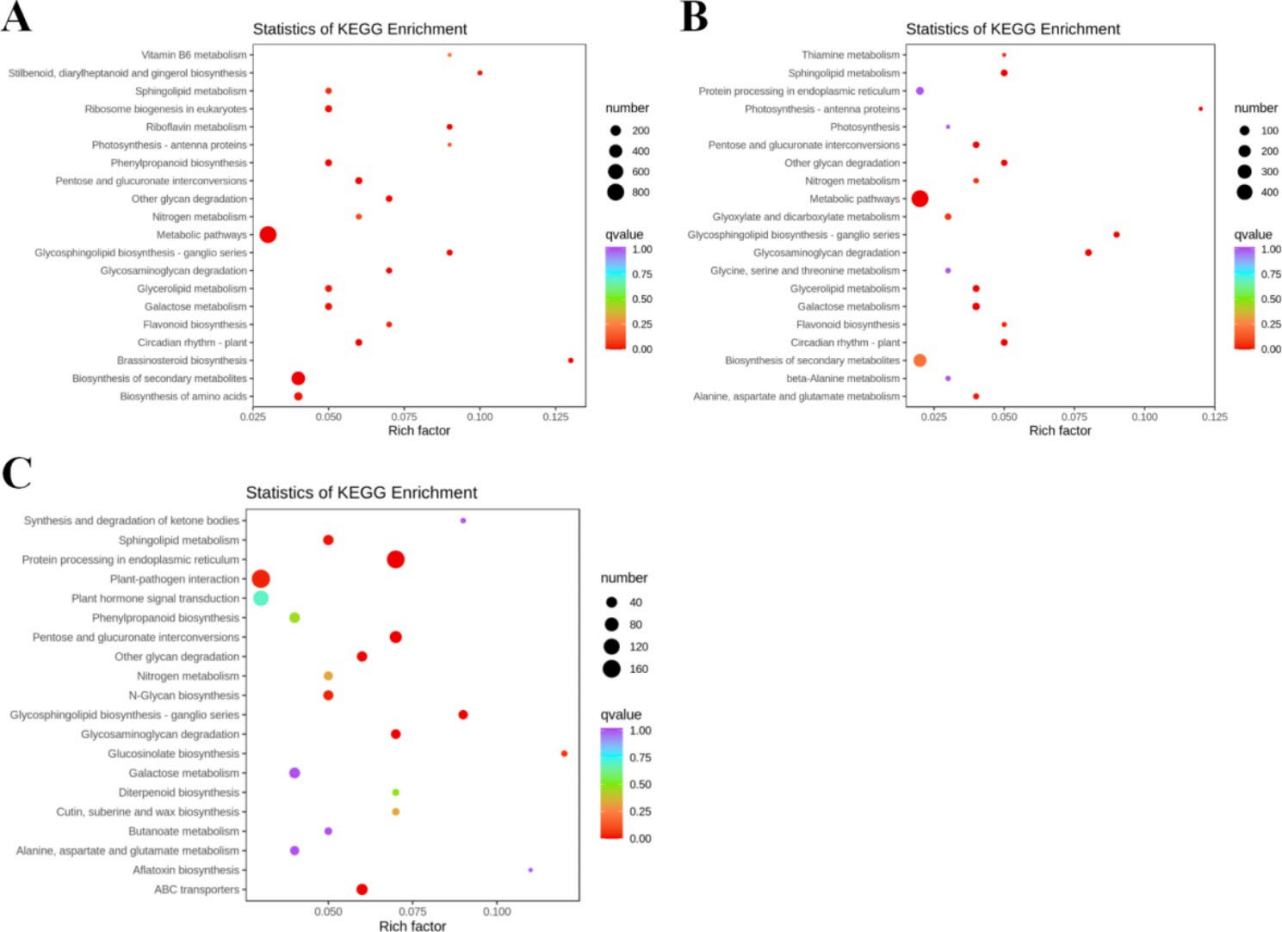
KEGG enrichment analysis. (A) T0 vs. T1. (B) T0 vs. T2. (C) T0 vs. T4

The top 20 KEGG enrichment pathways with the lowest Q values in the three comparisons were shown in Fig. 4. In T0 vs. T1, DEGs were significantly enriched in the “Phenylpropanoid biosynthesis”, “Flavonoid biosynthesis” and “Photosynthesis- antenna proteins” pathways. Similar to T0 vs T1, DEGs were also significantly enriched in the “Flavonoid biosynthesis” and “Photosynthesis - antenna proteins” pathways in T0 vs. T2. Significant enrichment was also identified in the “Protein processing in endoplasmic reticulum” pathway. In T0 vs. T4, DEGs were significantly enriched in the “plant hormone signal transduction”, “protein processing in endoplasmic reticulum “, “N-Glycan biosynthesis”, “protein export” and “phenylpropanoid biosynthesis”, etc. These results indicated that ALA could exert effects on complex biological pathways of *P. wutunensis* under the salt stress (Fig. 4).

Effect of exogenous ALA on jasmonic acid signalling in *P. wutunensis* under salt stress.

**Fig. 5:**
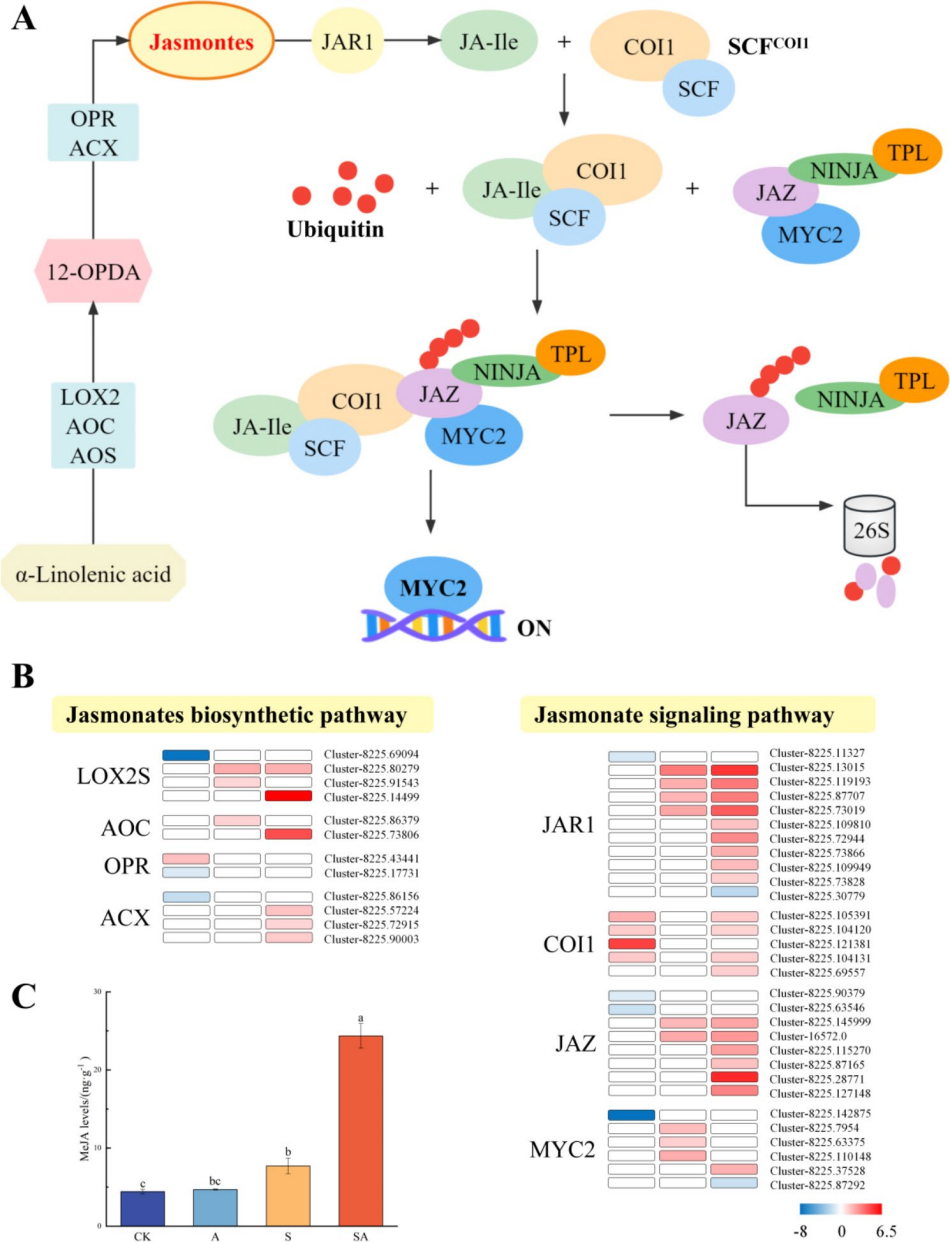
Jasmonic acid signaling-related pathways and MeJA content. (A) Jasmonic acid signaling-related pathways. (B) DEGs in Jasmonic acid signaling-related pathways. Red represents up-regulation, and blue represented down-regulation. (C) MeJA content of *P. wutunensis* seedlings under different treatments. Data were expressed as the mean ± standard error of three independent biological replicates. Different letters indicated significant differences of P < 0.05 according to Duncan test

As plants adapt to external stresses by altering the expression of certain hormone levels which in turn will regulate gene expression, plant hormone signaling pathways need to be analyzed. The results showed that exogenous ALA mobilized several hormonal signals in plants, such as abscisic acid, jasmonic acid and growth hormone, with the jasmonic acid signaling pathway being significantly activated. In contrast to T0, exogenous ALA treatment under salt stress up-regulated the expression of several genes in the jasmonic acid biosynthetic pathway and the signal transduction pathway (Fig. 5A and B). The synthesis of JAs is essentially a cascade of enzymatic reactions starting with the release of linolenic acid from the cell membrane as a substrate. The expression levels of genes encoding JA synthesis-related proteins such as lipoxygenase (LOX2S), allene oxide cyclase (AOC), 12-oxophytodienoic acid reductase (OPR3) and acyl-CoA oxidase (ACX) were increased, especially in T0 vs. T2 and T0 vs. T4. Almost all genes in the JA signaling pathway were induced by ALA, with 8 (4 up-regulated and 4 down-regulated), 9 (all up-regulated) and 22 (20 up-regulated and 2 down-regulated) DEGs in T0 vs. T1, T0 vs. T2 and T0 vs. T4 respectively. Under the ALA-treatment, in addition to jasmonic acid-amino synthetase (JAR1) and coronatine-insensitive protein 1 (COI1), which were significantly up-regulated, four MYC2, a core regulatory factor, were strongly induced by ALA to significantly higher expression levels. The expression levels of eight jasmonate ZIM domain-containing protein (JAZ) were also affected. These results indicated that the application of exogenous ALA positively regulated the accumulation of Jasmonates in *P. wutunensis* and activated the jasmonic acid signaling pathway, which was likely to be the key for ALA to improve salt-tolerance.

Jasmonic acid (JA) is an important hormone which regulates salt-tolerance in plants. Methyl jasmonate (MeJA) is a volatile ester form of JA that regulates the expression of genes associated with plant defense mechanisms in response to biotic and abiotic stresses. Therefore, a LC-MS/MS platform was used to assess whether the ALA-treatment affected the levels of MeJA in leaves of *P. wutunensis* under normal and salt-stressed conditions. Exogenous spraying of ALA under normal conditions had no significant effect on MeJA levels in *P. wutunensis*. However, the MeJA production was triggered by ALA with the salt treatment. Under salt stress, exogenous ALA significantly increased MeJA levels by 216% compared to the group S, indicating that MeJA accumulation might be induced by ALA (Fig. 5C).

**Fig. 6:**
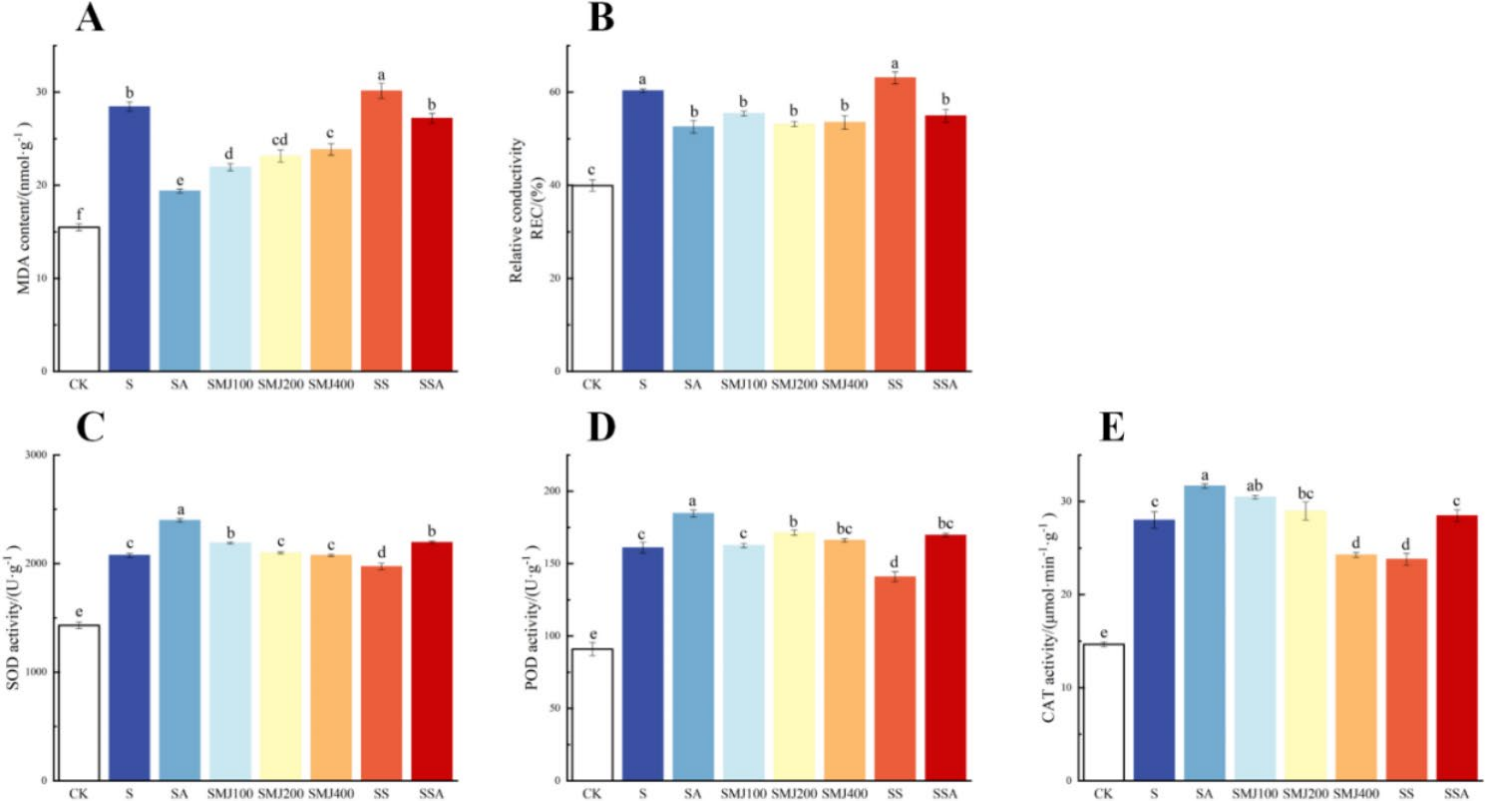
The MDA content, the REC and antioxidant enzyme activities of *P. wutunensis* seedlings under different treatments. (A) MDA content; (B) REC; (C) SOD activity; (D) POD activity; (E) CAT activity. CK: normal growth; S: salt stress; SA: salt stress + ALA; SMJ100: salt stress + 100 µM MeJA; (5) SMJ200: salt stress + 200 µM MeJA; (6) SMJ400: salt stress + 400 µM MeJA; (7) SS: salt stress + 200 µM SHAM; (8) SSA: salt stress + 200 µM SHAM + ALA. Data were expressed as the mean ± standard error of three independent biological replicates. Different letters indicated significant differences of P < 0.05 according to Duncan test

To investigate the role of MeJA in ALA-induced salinity tolerance, seedlings were pretreated with 200 µM salicylhydroxamic acid (Jasmonates biosynthesis inhibitor, SHAM) and then analyzed for changes in the MDA content, the relative conductivity and antioxidant enzyme activities. The MDA content and REC were significantly higher in all plants under salt stress compared to CK, indicating that salt stress caused membrane lipid damages in the plants. Under salt stress, plants in the SA and SMJ groups showed significantly lower MDA content and REC compared to the group S, indicating that both MeJA and ALA alleviated the membrane lipid damage caused by salt stress. SHAM which should inhibit endogenous MeJA biosynthesis had no significant effect on the REC of plants in the group SS compared to the group S. On the other hand, plants in the group SSA underwent a significant increase in the MDA content compared to plants in the SA and SMJ groups, indicating that the ability of ALA to alleviate the membrane lipid damage was affected after inhibition of endogenous MeJA synthesis (Fig. 6A and B).

Salt stress significantly increased SOD, POD and CAT activities compared to CK. MeJA treatment increased SOD, POD and CAT activities compared to the group S, with a better effect at 200 µM, while SHAM-treatment significantly reduced the antioxidant enzyme activities. Under salt stress, the antioxidant enzyme activities of plants in the group SSA were significantly reduced compared to the group SA, indicating that the antioxidant capacity of ALA was affected by the inhibition of endogenous MeJA synthesis. These results suggested that the exogenous ALA-enhancement of salt-tolerance in *P. wutunensis* was mediated by the endogenous MeJA production. (Fig. 6C, D and E).

Effect of exogenous ALA on protein processing-related to the endoplasmic reticulum pathways of *P. wutunensis* under salt stress.

**Fig. 7:**
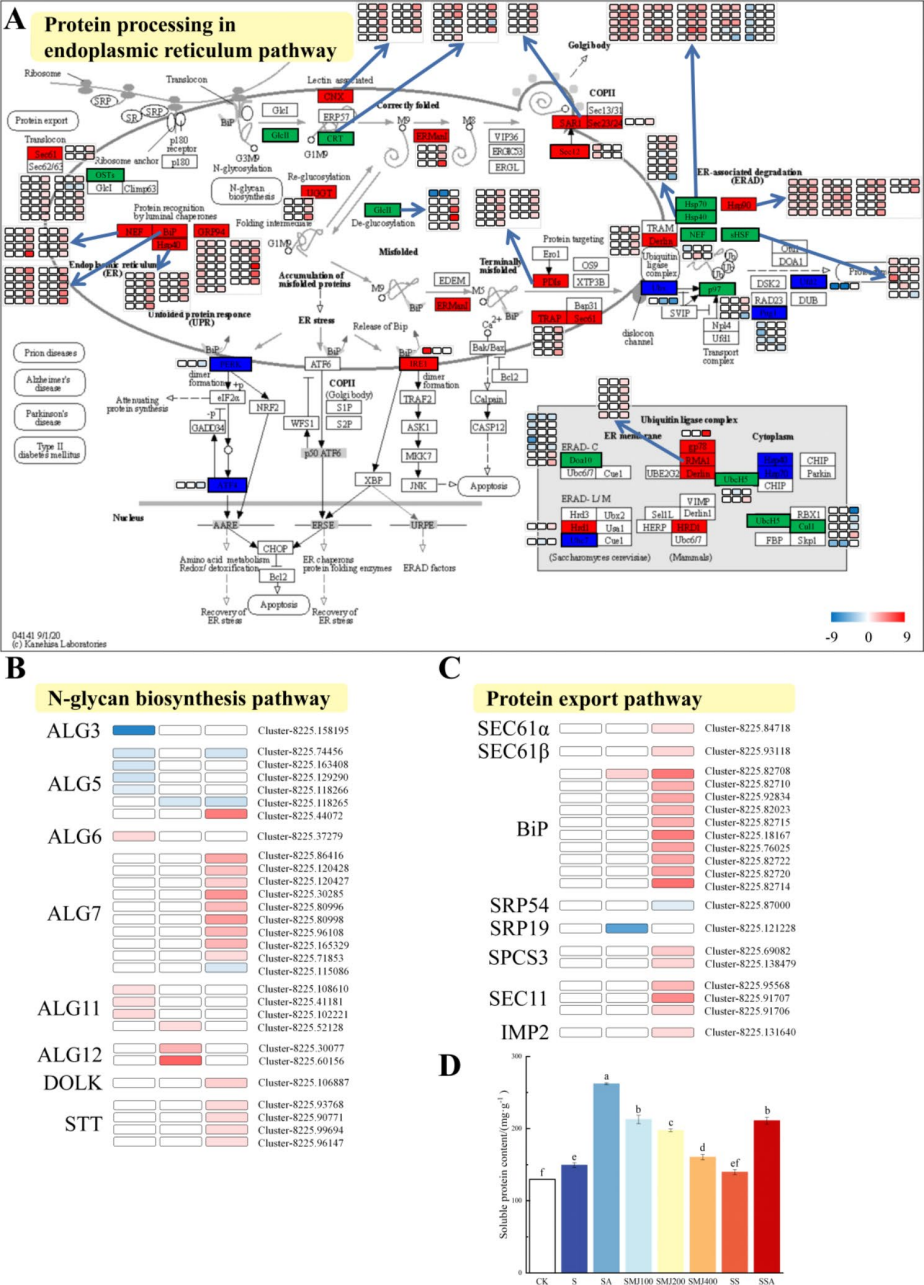
Protein processing-related in the endoplasmic reticulum pathways and soluble protein content. (A) Protein processing in endoplasmic reticulum pathway. Red represented up-regulation, blue represented down-regulation, green represented both up- and down-regulation. (B) DEGs in the N-glycan biosynthesis pathway. (C) DEGs in the protein export pathway. (D) Soluble protein content. Data were expressed as the mean ± standard error of three independent biological replicates. Different letters indicated significant differences of P < 0.05 according to Duncan test

According to the KEGG pathway analysis, ALA-treatment up-regulated the expression levels of a substantial number of genes in the protein processing in endoplasmic reticulum of *P. wutunensis* under thevsalt stress (Fig. 7A). There were 51 (33 up-regulated and 18 down-regulated), 54 (41 up-regulated and 13 down-regulated) and 160 (145 up-regulated and 15 down-regulated) DEGs in the protein processing in endoplasmic reticulum pathway in T0 vs. T1, T0 vs. T2 and T0 vs. T4, respectively. These genes were annotated to encode enzymes which covered almost the entire spectrum of protein processing in endoplasmic reticulum, including mannosyl-oligosaccharide alpha-1,3-glucosidase (GlcII), calnexin (CNX), calreticulin (CRT), mannosyl-oligosaccharide alpha-1,2-mannosidase (ERMan I), GTP-binding protein (SAR1), UDP-glucose:glycoprotein glucosyltransferase (UGGT), oligosaccharyltransferase complex subunit (OSTs) and protein disulfide-isomerase (PDIs), as well as various molecular chaperones such as Heat shock protein 70 (Hsp70), Heat shock protein 90 (Hsp90), nucleotide exchange factors (NEFs), binding protein (BiP) and Glucose-related protein 94 (GRP94), all of which were up-regulated. At the same time, sub-pathways associated with endoplasmic reticulum processing such as N-glycan biosynthesis and protein export (Fig. 7B and C) were also significantly enriched in the similar pattern. In T0 vs. T4 there were 30 (26 up-regulated and 4 down-regulated) and 19 (18 up-regulated and 1 down-regulated) DEGs enrichments in N-glycan biosynthesis and protein export, respectively. DEGs such as N-acetylglucosamine-1-phosphate transferase (ALG7) and dolichol kinase (DOLK) were significantly up-regulated in N-glycan biosynthesis. In the protein export pathway, DEGs such as protein transport protein (Sect. 61), signal peptidase (Sect. 11) and BiP were significantly up-regulated. The up-regulation of key genes in the N-glycan biosynthesis and protein export pathways could accelerate the transporting efficiency of unfolded nascent peptides to the endoplasmic reticulum via the Sect. 61 transport system, while providing sufficient N-glycans for protein glycosylation modifications. These results showed that ALA largely influenced the protein processing in endoplasmic reticulum pathway of *P. wutunensis* under salt stress by accelerating the folding efficiency of correctly folded proteins with helping proteins and directing the degradation of misfolded proteins.

Soluble proteins are important osmoregulatory molecules protecting the vital substances of cells and biofilms. Soluble proteins include enzymes that are involved in various metabolisms and also play important roles in the salt-resistance of plants. Additionally, the soluble protein content also reflects the efficiency of protein folding in the endoplasmic reticulum. In this study, the content of soluble proteins of plants in the group S was increased compared to the group CK. Both MeJA and ALA treatments significantly increased the soluble-protein content compared to the group S, while inhibition of MeJA synthesis with SHAM decreased it. Under salt stress, the soluble-protein content of plants in the group SSA was significantly lower than those in the SA / SMJ group, indicating that the ability of ALA to increase protein folding efficiency was hindered by the inhibition of endogenous MeJA synthesis (Fig. 7D).

Effect of exogenous ALA on the flavonoid biosynthesis pathway in *P. wutunensis* under salt stress.

**Fig. 8:**
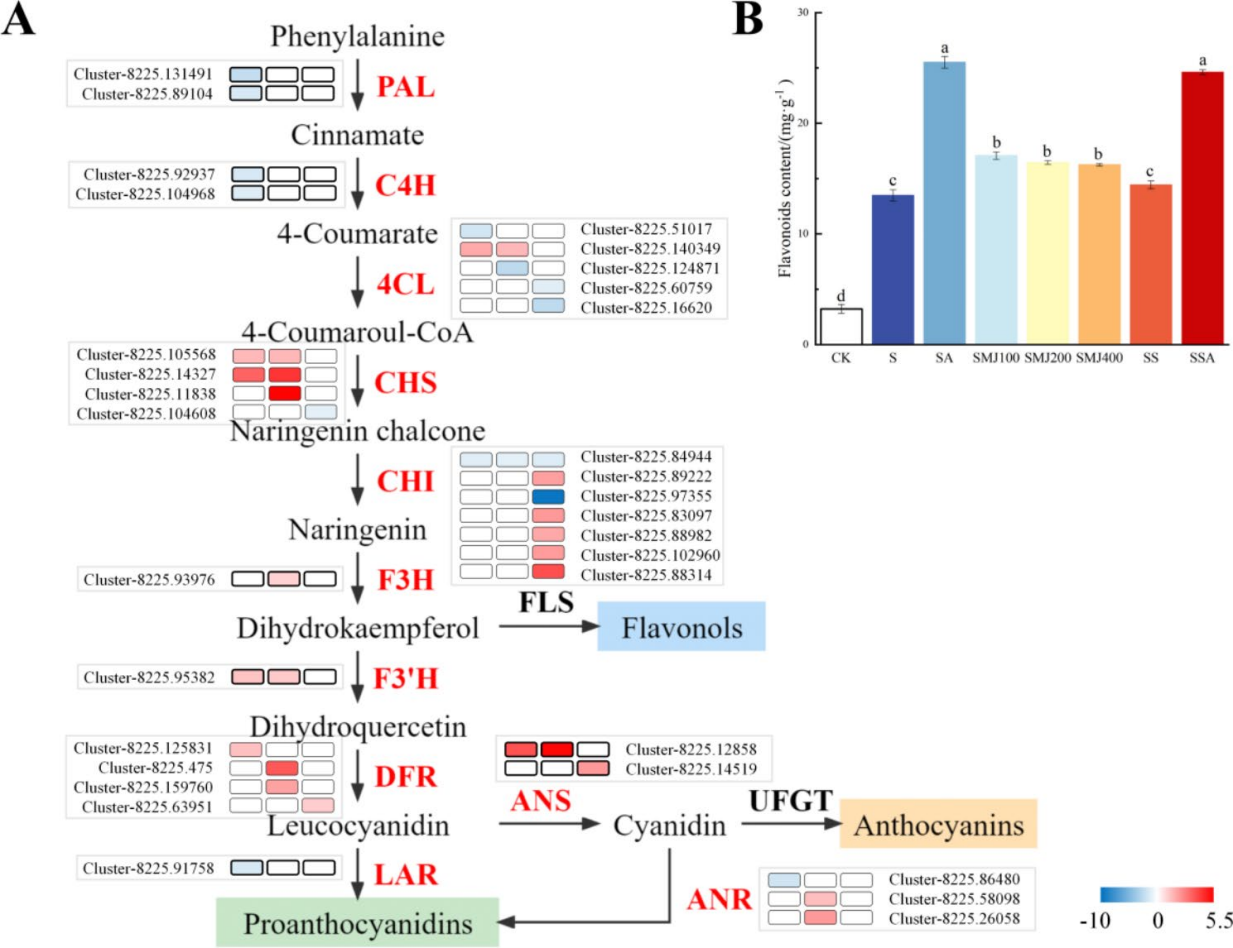
Flavonoid biosynthesis pathway and flavonoid content. (A) Flavonoid biosynthesis pathway. Red represented up-regulation, blue represented down-regulation. (B) Flavonoid content. Data were expressed as the mean ± standard error of three independent biological replicates. Different letters indicated significant differences of P < 0.05 according to Duncan test

To investigate the effect of exogenous ALA on the flavonoid biosynthetic pathway of *P. wutunensis* under salt stress, relevant DEGs were screened and analyzed (Fig. 8A). In ALA-treated and salt-stressed plants, the expression levels of phenylalanine ammonia-lyase PAL, cinnamate 4-hydroxylase C4H and 4-Coumarate-CoA ligase 4CL (genes belonged to the early stages of the flavonoid synthesis pathway) were slightly reduced, but the transcription of chalcone synthase CHS (gene catalyzed the conversion of coumaroyl-CoA and malonyl-CoA together into chalcone) was significantly up-regulated by more than four times of the control. Dihydroflavonol 4-reductase (DFR) and Anthocyanin Synthase (ANS) are key enzymes in the flavonoid pathway for the synthesis of anthocyanins and proanthocyanidins. Four DFR and two ANS homolog genes were found to be upregulated in this study. In addition, the expression levels of other flavonoid biosynthesis-related genes including three CHS, five chalcone isomerase (CHI), one naringenin 3-dioxygenase (F3H) and one flavonoid-3’hydroxylase (F3’H) were also found increased. Flavonoid compounds such as flavonols, anthocyanins and proanthocyanidins are antioxidants and flavonoid compounds might act as important mediators of ALA, enhancing the scavenging of ROS and mitigating oxidative damage caused by salt stress.

Flavonoids, which possess oxygen-radical scavenging functions, participate in the integrated plant defense system to reduce cell damages from adverse stresses and thus to protect cells. In order to verify the relationship between ALA, MeJA and flavonoid compounds, the flavonoid content was examined under different treatments. Salt stress could increase the flavonoid content in treated plants compared to CK plants. Flavonoid content was significantly increased in plants treated with JA and especially with ALA compared to the group S. Compared to the group SS, the ALA-treatment with JA-biosynthesis inhibition still increased the flavonoid content to a similar level as in the only ALA-treated plants. This observation suggested that ALA would increase the flavonoid content through inducing the accumulation of MeJA in *P. wutunensis* but there might well be other ALA-induced signaling pathways existed besides the MeJA pathway to perceive and resist salt stress (Fig. 8B).

Quantitative RT-PCR validation.

**Fig. 9:**
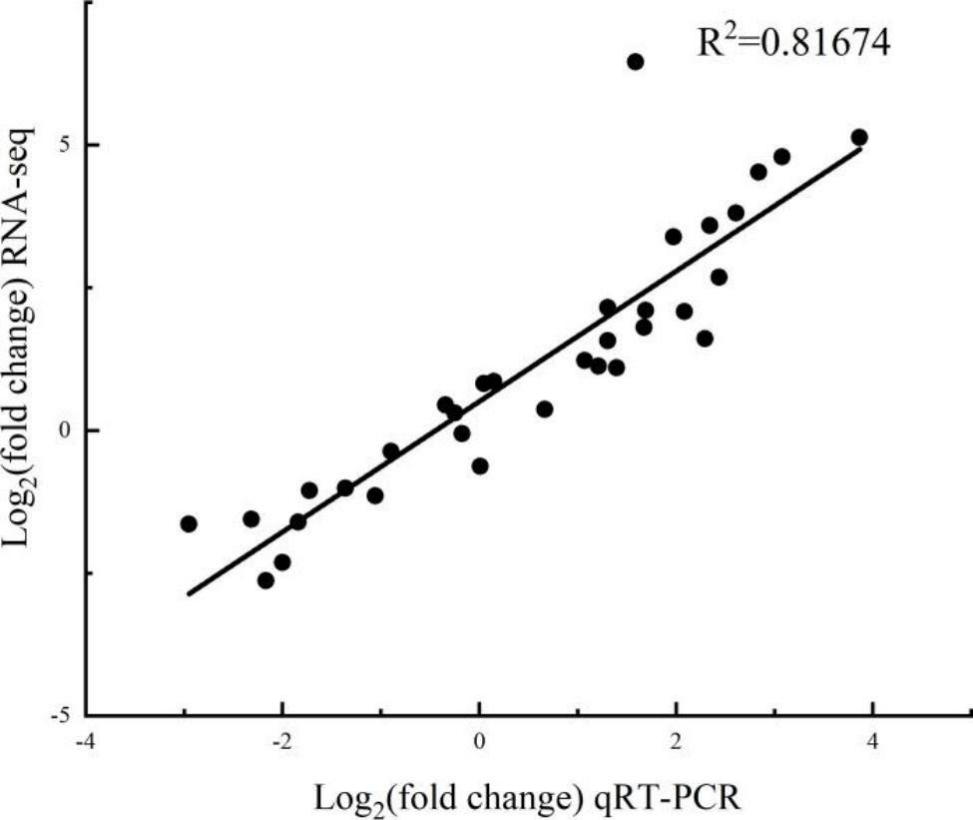
Correlation between RNA-Seq expression profile and qRT-PCR results

To test the accuracy of our RNA-Seq data, quantitative RT-PCR (qRT-PCR) was performed on 11 DEGs included *MYC2*, *JAR1*, *JAZ*, *AOC*, *LOX2S*, *OPR*, *BIP*, *Doa10*, *CHI*, *DFR* and *PAL*, which were selected from the jasmonic acid biosynthetic and the signal transduction pathway, protein processing in endoplasmic reticulum pathway and flavonoids biosynthesis pathway. Consistently, the qRT-PCR results exhibited the same trend which were correlated with the RNA-Seq data (R^2^ = 0.81674), thus confirming the confidentiality of the RNA-Seq data (Fig. 9).

## Discussion

5-Aminolevulinic acid (ALA) is a multi-functional plant-growth regulator which regulates plant growth and development and induces adaptive responses to biotic and abiotic stresses [8]. Cengiz and Muhammad (2020) found that 5-aminolevulinic acid could induce salt-tolerance in *Zea mays* by attenuating oxidative stress [31]; Wu et al. (2019) showed that ALA attenuated the damage caused by NaCl by enhancing the AsA-GSH pathway in cucumber seedlings [32]; Wu et al. (2019) suggesting that ALA induces H_2_O_2_ accumulation in roots, which mediates Na^+^ transporter gene expression and more Na^+^ retention in roots, thereby improving strawberry salt tolerance [33]. In the present study, ALA-treated *P. wutunensis* seedlings had shown better growth than untreated seedlings under salt-stress conditions and were able to maintain relatively lower ROS accumulation and higher activities in antioxidant enzymes. In addition, expression of some antioxidant system-related genes, aquaporin gene and Na^+^ transmembrane transporters were also significantly affected by ALA-treatment in salt-stressed plants. Therefore, treatment with ALA would pose positive effects on *P. wutunensis* growth under salt stress and our findings were consistent with the results of Jiao et al. (2021) [34].

Plant hormones play crucial roles in coping with environmental stresses [35]. Phytohormones often require complex signal networking in combination with other signaling pathways to perform their proper functions. Therefore, the understanding on plant hormone signaling is a prerequisite for investigating plant defense mechanisms against stressful conditions [36]. Waterlogging tolerant soybean varieties mitigate the adverse effects of waterlogged condition on adventitious roots growth and development by regulating hormone levels [37]. Rice promotes fluoride tolerance by reducing abscisic acid accumulation and increasing its melatonin and gibberellic acid levels [38]. In the present study, transcriptome analysis showed that exogenous ALA modulated several known hormonal signals among which the biosynthetic and signal-transduction pathways of jasmonic acid were significantly activated.

The free-acid jasmonic acid (JA) and JA-derivatives collectively are known as jasmonates (JAs) which are important lipid-derived cellular regulators [39]. JAs are also key signaling factors in various developmental and defensive processes in plants especially playing an important role in inducing the salt-stress responses [40]. Jasmonic acid can effectively protect wheat seedlings from salt stress by inhibiting salt stress-induced excess reactive oxygen species through increasing the activities of antioxidant enzymes and the concentration of antioxidant substances [41]; foliar sprays of JA mitigated the negative effects of salt on the growth and metabolism in two soybean species by improving ion homeostasis and increasing photosynthetic pigment content [42]; and JA signaling pathways play an important role in the response of salt-tolerant sweet potato ND98 to salt stress [43]. These evidences suggest that JAs are highly relevant to capabilities of plants in resisting salt stress. Moreover, JAs are considered to be the central signals in plant hormone-signaling networks, regulating the balance between plant growth and defense [44]. In this study, the expression of important genes in the jasmonic acid biosynthetic pathway such as LOX2S, AOC, OPR3 and ACX was increased and the level of MeJA was also increased in ALA-treated plants under salt stress, significantly higher than those in the non-ALA-treated group. Exogenous MeJA exerted similar salt stress alleviating effect as ALA. And the stress-alleviating ability of ALA was affected by the SHAM inhibition of endogenous MeJA. These results suggested an interactive relationship between ALA and JAs in which the mediation through MeJA was critical for the ALA-induced salt-stress perception and tolerance in *P. wutunensis*. These findings were similar to those in Liu et al. (2019c) and Anwar et al. (2018) [45, 46].

Methyl jasmonate (MeJA) is a methyl derivative of JA that leads to the transcriptional activation of many secondary metabolic pathways and mediates a variety of plant physiological processes [47]. MeJA can activate or repress the activities of corresponding transcription factors, which in turn regulate the expression of key enzyme genes related to plant secondary metabolism and ultimately the synthesis of secondary metabolites [48]. MeJA can induce the expression of genes related to not only the JA biosynthetic pathway but also the JA signaling pathway [49]. Furthermore, it has been shown that MeJA treatment can activate the jasmonic acid signaling pathway more effectively than the same concentration of JA, and the transcriptional induction of jasmonic acid-responsive genes is faster and the expression is higher under MeJA treatment than JA treatment [50]. In salt-stressed *P. wutunensis*, JAR1, which promotes the formation of the highly bioactive JA-Ile from JA, the jasmonic acid receptor F-box protein COI1 and the hormone coregulator MYC2 were all induced to be up-regulated after the application of ALA, and the expression level of the ZIM structural domain protein (JAZ) was also affected. These changes were most likely due to the activation of jasmonic acid signaling channels by ALA-induced production of JAs. Notably, MYC2 has been identified as a major node in the interaction of jasmonic acid with other hormone signaling pathways [51], acting as a master switch for the regulation of jasmonic acid-mediated biosynthesis of secondary metabolites [52]. Therefore, MYC2 was a crucial transcription factor in the ALA-induced JA signaling pathway.

In our results, not only did the expression of genes encoding jasmonic acid signaling components differ with the ALA-treatment, but the expression of genes encoding signaling components of other major plant hormones such as abscisic acid, cytokinin, growth hormone, oleuropein lactone and ethylene also changed. Some of these genes encoded components related to the interaction of JAs with other hormones, such as DELLA and JAZ associated with JA-GA [53], ARF and IAA associated with JA-Auxin [54], ERF1 and EIN3 associated with JA-ET [55]. The expression of genes such as PYR/PYL associated with JA-ABA was significantly affected [56]. It is known that the output of JA signaling depends on complex cross-talks between JAs and other hormones and ultimately mediates the transcriptional regulation of secondary metabolic pathways [57]. And our findings concurred to this understanding.

Adverse stress can often lead to the accumulation of unfolded or misfolded proteins in the endoplasmic reticulum. To alleviate stress, the endoplasmic reticulum will restore homeostasis through the unfolded protein response (UPR), the endoplasmic reticulum associated degradation (ERAD), and the regulation of translation-related gene expression [58]. ERAD is essential for plants to overcome salt stress [59], helping to eventually degrade proteins that are not properly folded in the endoplasmic reticulum. Salt stress stimulates NEFs to tightly regulate BiP activity causing a hypersensitive response to endoplasmic reticulum stress and thus affecting plant growth [60]. These findings suggest the plausibility of that plants will respond to salt stress by enhancing protein folding capacity through the expression of endoplasmic reticulum-related genes. In the present study, the ALA-treatment up-regulated the expression of most genes in the endoplasmic reticulum protein pathway in salt-stressed plants, and these genes encoded enzymes that covered almost the entire process of protein processing in endoplasmic reticulum. The ALA-treatment could accelerate the degradation of correctly folded and misfolded proteins. Furthermore, the soluble protein content in ALA-treated plants with inhibition of endogenous JAs production was lower than those without inhibition, suggesting that the jasmonate signaling pathway was closely related to endoplasmic reticulum stress. Xu et al. found that in Arabidopsis, the JA signaling pathway plays an important role in the induction of chaperone protein genes and the IRE1-bZIP60 pathway [61]. Similarly, ALA might promote protein folding efficiency in the endoplasmic reticulum by activating the jasmonic acid signaling pathway, which in turn would improve the salt-tolerance in *P. wutunensis*.

Flavonoids are a group of secondary metabolites that are widely distributed in plants [62]. They are classified into several major subgroups such as anthocyanins, proanthocyanidins, flavonols, flavones and isoflavones [63]. Flavonoids play important biological roles in plant development and defense and are considered to be the main antioxidants against oxidative damage and necessary for plant adaptation to biotic and abiotic stresses. In the present study, the expression levels of *CHS*, *CHI*, *F3H*, *F3’H*, *DFR* and *ANS*, which are associated with flavonoid biosynthesis, were significantly elevated in plants treated with ALA under salt stress. Our metabolite quantification also showed that the flavonoid content was elevated, in agreement with the findings of Farhadi et al. (2022) [64]. Flavonoid biosynthesis is regulated by a variety of factors, including temperature, moisture, light and hormonal signals [65]. The effects of plant hormones such as jasmonic acid, abscisic acid, auxin, ethylene, cytokinin and gibberellin on flavonoid accumulation have been extensively studied [66]. Zhang et al. found that salt signals in *Dendrobium* leaves could induce JA biosynthesis, and JA acted as a signaling molecule to promote flavonoid biosynthesis [67]. Both exogenous MeJA and ALA could increase the flavonoid content in *P. wutunensis* leaves under salt stress, while the ability of ALA to enhance flavonoid accumulation was reduced after treatment with SHAM. Flavonoids have antioxidant activities, and the process of ALA increasing flavonoid content in *P. wutunensis* seedlings which could be mediated by MeJA signaling may enhances the scavenging of ROS and attenuates oxidative damage caused by salt stress.

Transcription factors (TFs) can interact with cis-elements within promoters to regulate the transcription of downstream genes and have an important role in plant resistance to abiotic stresses [68]. It was found that ALA-treatment induced gene expression of many different families of TFs that were shown to be regulators of the biosynthesis of different secondary metabolites, such as AP2/ERF, bHLH, MYB, NAC and WRKY, which agreed with previous studies [69]. These transcription factor families could act as central regulators and molecular switches in the complex salt stress signaling network by activating or repressing the specific expression of a gene or group of genes, whose products in turn would control the expression of downstream genes or directly protect plants from salt stress [70]. The specific regulatory roles of these TFs in improving salt-tolerance in plants and the mode of regulation still remained to be further investigated. Our results of qRT-PCR analysis verified the authenticity of the expression patterns obtained in the present transcriptome analysis. In the future, functional characterization of key node genes in the pathway should be performed by construction of transgenic *P. wutunensis*.

**Fig. 10:**
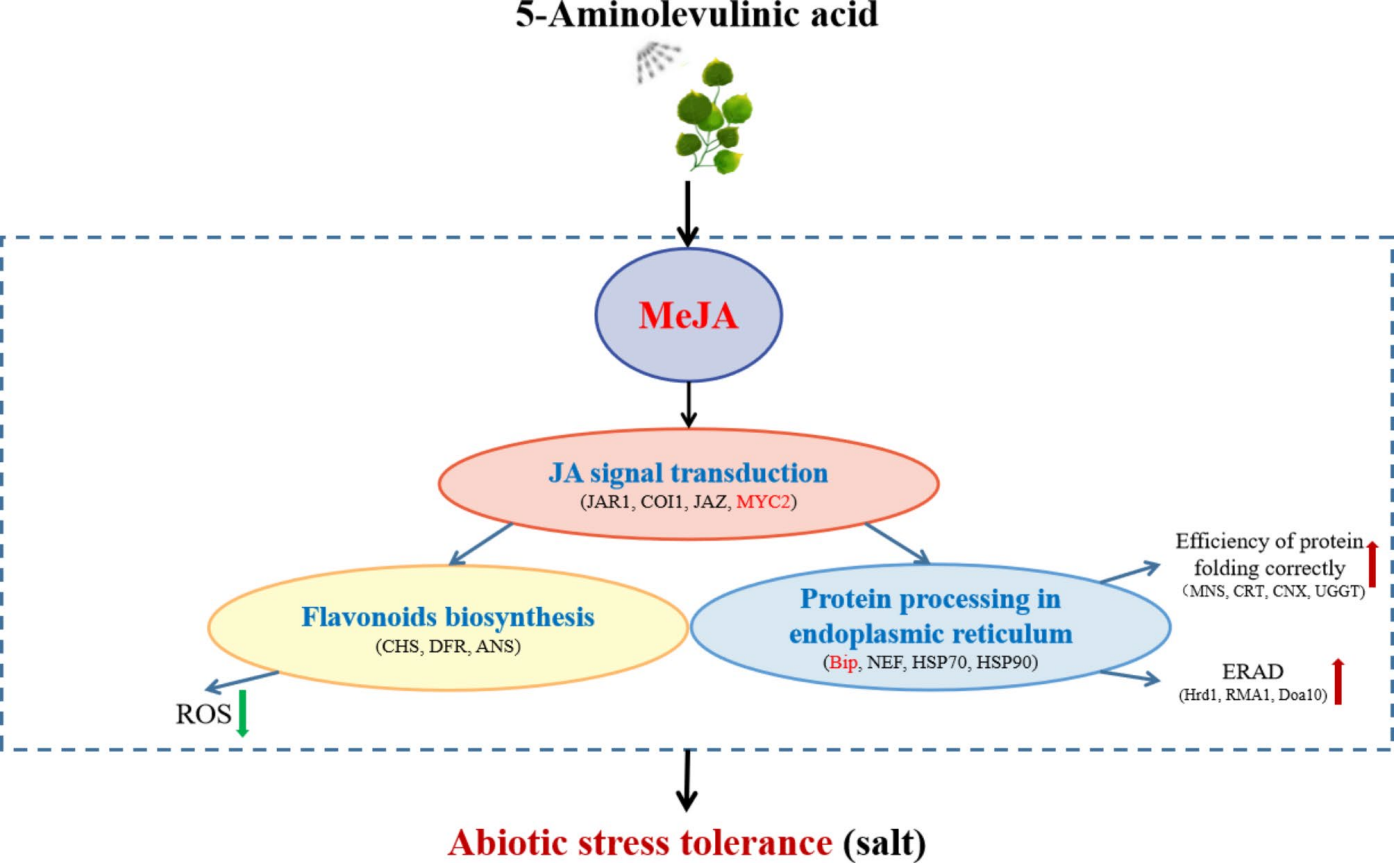
ALA improves salt tolerance in *P. wutunensis* by affecting gene expression levels

## Conclusion

In conclusion, exogenous ALA induced the accumulation of MeJA in *P. wutunensis* under salt stress. Elevated MeJA activated the jasmonic acid signaling pathway, enhanced protein folding efficiency in the endoplasmic reticulum and induced flavonoid biosynthesis all of which helped with maintaining the osmotic homeostasis and protein stabilities and improving oxidative defense, thereby enhancing the salt-tolerance in *P. wutunensis* (Fig. 10). In this study, a conceptual framework was proposed that MeJA might act as an important mediator of ALA-enhancement of plant tolerance to multiple stresses, which provided new insights for future studies on the molecular mechanisms underlying the multiple functions of ALA.

## Materials and methods

Plant materials and growing conditions.

The material used for the experiments was cultivated from 8-year-old branches of *P. wutunensis* grown by the Fuxin Forestry Bureau. Good, disease-free annual branches of the *P. wutunensis* were selected, and cuttings of 20 cm down the middle were placed in pots (27 cm diameter). The substrate is a 1:1 mixture of charcoal and sand. Water once every 3 days during the cultivation period. After 53 days (early lignification) of incubation of cuttings in the same conditions in a solarium (average temperature of 25.0 ± 2 °C and relative humidity of 60 ± 5%), seedlings of approximately uniform growth were selected for testing.

To investigate the effect of exogenous ALA on *P. wutunensis* under salt stress, plants were divided into two groups and watered with 800 mL of deionized water or 200 mmol•L^− 1^ NaCl, respectively. At 20:00 on the 8th day of watering the above two groups of plants were subdivided into two groups, with one group of leaves sprayed with deionized water and the other with 50 mg•L^− 1^ ALA. It is advisable to spray the front and back of the leaves wet and just drop liquid. This means a total of 4 treatments: (1) CK: watering with deionized water + spraying with deionized water; (2) A: watering with deionized water + spraying with ALA; (3) S: watering with NaCl + spraying with deionized water; (4) SA: watering with NaCl + spraying with ALA. Three independent biological replicates for each treatment.

To further investigate the molecular mechanism of exogenous ALA in alleviating salt stress in *P. wutunensis*, leaves 3 to 5 of plants in the group SA were collected on days 0, 1, 2 and 4 of the ALA treatment, noted as T0, T1, T2 and T4 respectively (T0 corresponds to the salt stress performed), and 3 independent biological replicates of each treatment were immediately frozen in liquid nitrogen and placed in a -80℃ refrigerator pending subsequent RNA-Seq analysis.

To investigate whether exogenous ALA could induce MeJA accumulation in the plants, leaves from day 1 of the ALA treatment were immediately frozen in liquid nitrogen and placed in a -80℃ refrigerator for subsequent assays of MeJA content.

To evaluate the role of MeJA in ALA-induced salt tolerance, the plant was pretreated with 200 µM salicylhydroxamic acid (SHAM) 24 h before salt stress, which can inhibit jasmonic acid family biosynthesis by inhibiting the lipoxygenase (LOX). Eight treatments were set up: (1) CK; (2) S: salt stress; (3) SA: salt stress + ALA; (4) SMJ100: salt stress + 100 µM MeJA; (5) SMJ200: salt stress + 200 µM MeJA; (6) SMJ400: salt stress + 400 µM MeJA; (7) SS: salt stress + 200 µM salicylhydroxamic acid (SHAM); (8) SSA: salt stress + 200 µM SHAM + ALA. In this case, spraying with ALA / MeJA was carried out on day 8 of salt stress. It is advisable to spray the front and back of the leaves wet and just drop liquid. MDA content, REC, antioxidant enzyme activity, soluble protein content and flavonoid content were measured on day 4 of the ALA / MeJA treatment.

Physiological index measurements and histochemical staining.

Conductivity was measured using a conductivity meter (Orion-METTLER-FE30K) at room temperature (24 °C) and calculated as described by Xu et al. (2019a) [71]. MDA, soluble protein and flavonoid compounds were measured separately using a commercial kit (Suzhou Michy Biomedical Technology Co., Ltd) and antioxidant enzyme activities including SOD, POD and CAT were assessed.

To detect superoxide accumulation, hydrogen peroxide accumulation and cell death, leaves were permeabilised with nitrotetrazolium blue chloride (NBT), 3,3’-Diaminobenzidine (DAB) and Evans blue (EB) according to methods described in detail by Zhang et al. (2011) and Kim et al. (2003) [72, 73].

MeJA content assay.

Phytohormones contents were detected by MetWare (http://www.metware.cn/) based on the AB Sciex QTRAP 6500 LC-MS/MS platform [74].

Transcriptome sequencing and data analysis.

Twelve samples (4 treatments × 3 biological replicates) were subjected to RNA-seq assays. Total RNA was extracted using TRIzol reagent (Invitrogen, Carlsbad, CA, USA) according to the manufacturer’s instructions. The quality of RNA was assessed by Agilent 2100. Sequencing was performed using the method described by Wang et al. (2015) [75]. Briefly, RNA concentrations were measured by a Qubit 2.0 Flurometer (Life Technologies, USA). cDNA libraries for sequencing were prepared using the NEBNext Ultra RNA LibraryPrep Kit.

After the libraries were constructed, the libraries were tested for quality. After the results of the assay met the requirements, the different libraries were pooled according to the target downstream data volume and then sequenced using the Illumina HiSeq platform. Reads with adapters, sequences with more than 10% unknown nucleotides (N) and reads with a quality level below 50% (Q value ≤ 20) were first removed from the dataset. After obtaining the clean reads, Trinity was used to splicing the clean reads and the spliced transcript sequences were used as reference sequences for subsequent analysis. HTSeq v0.6.1 is used to calculate the read counts mapped to each gene. The FPKM for each gene is then calculated based on the length of the gene and the read counts are mapped to that gene. Differential expression analysis between sample groups was performed using DESeq2 to obtain differentially expressed gene sets between the two biological conditions [76]. The screening criteria for differential genes were |log_2_FoldChange| ≥ 1 and FDR < 0.05. The functions of DEGs were annotated by GO function and KEGG pathway analysis through the OmicShare tool (https://www.genome.jp/kegg).

Quantitative real-time polymerase chain reaction (qRT-PCR) analysis.

To verify the accuracy of RNA-seq sequencing, 11 DEGs presumed to be associated with ALA-induced tolerance to salt stress were randomly selected for qRT-PCR validation. The Action20 gene was used as reference gene. Gene-specific primers were designed using Primer Premier5.0. QRT-PCR was performed on the ABI/Thermo Fisher (QuantStudioTM 1 Plus System, USA). Each gene was analysed in 3 biological samples and 3 replicates were performed for each biological sample.

Data statistics.

Analysis of variance (ANOVA) was performed using IBM SPSS Statistics 22. Plots were made using Origin 2018.

## Electronic supplementary material

Below is the link to the electronic supplementary material.

Supplementary Material 1

Supplementary Material 2

Supplementary Material 3

Supplementary Material 4

Supplementary Material 5

## Data Availability

Raw data was deposited in NCBI database under SRA accession: PRJNA909441 (https://www.ncbi.nlm.nih.gov/sra/PRJNA909441).
