# Supplementary material for: MeJA-mediated enhancement of salt-tolerance of Populus wutunensis by 5-aminolevulinic acid
Source: BMC Plant Biol. 2023 Apr 6;23:185. doi: 10.1186/s12870-023-04161-7 (PMC10077631; doi:10.1186/s12870-023-04161-7)
Supplement: Supplementary file 1 — Supplementary Material 1 [file 12870_2023_4161_MOESM1_ESM.docx]

**
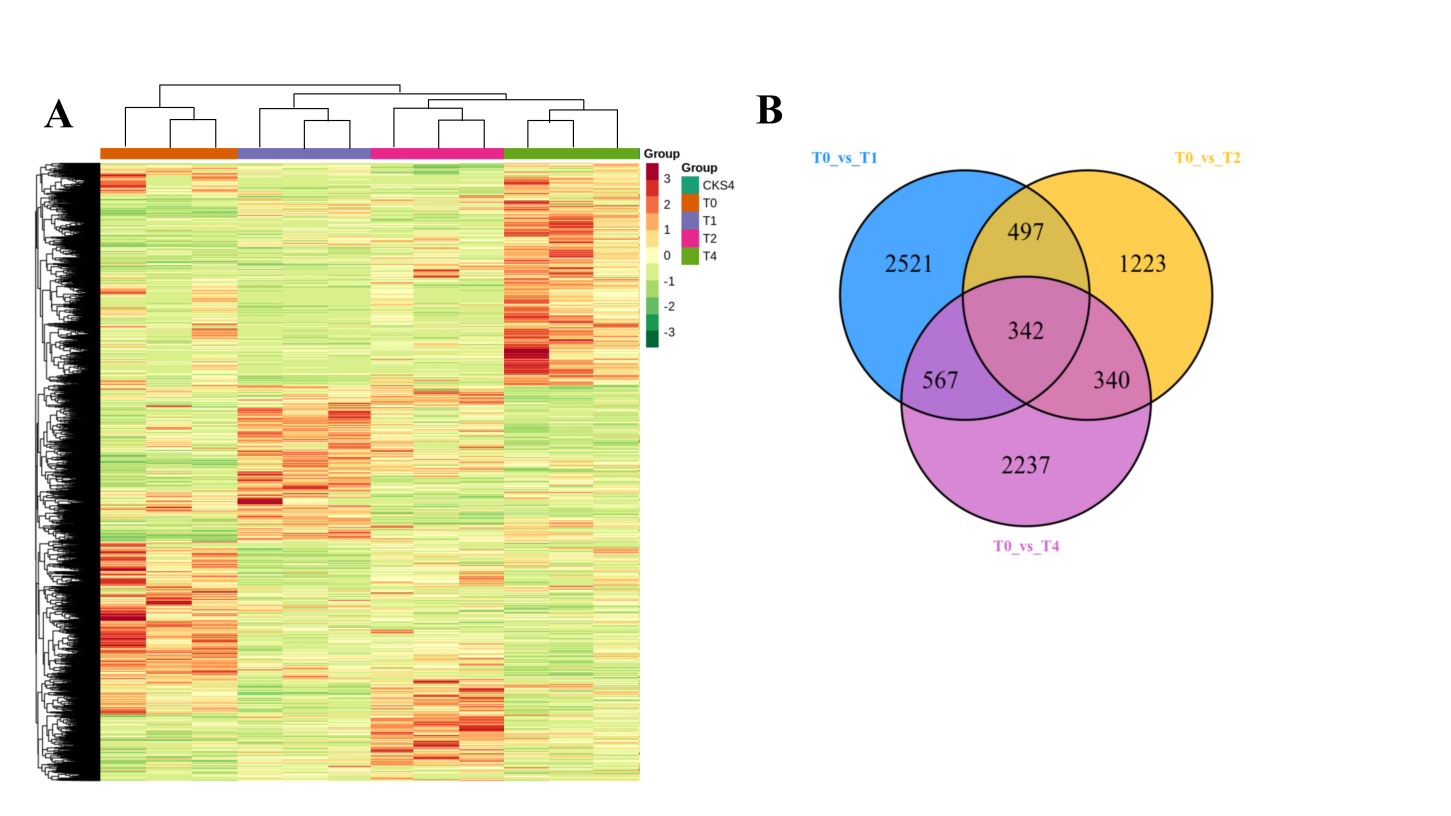
Fig. S1** Differentially expressed gene statistics. (A) Heat map of clustering of all differential genes. Horizontal coordinates indicated sample names and hierarchical clustering results, vertical coordinates indicated differential genes and hierarchical clustering results. Red indicated high expression, green indicated low expression. (B) Venn diagrams for DEGs in T0 vs T1, T0 vs T2 and T0 vs T4.
