# Supplementary figures and images for: MeJA-mediated enhancement of salt-tolerance of Populus wutunensis by 5-aminolevulinic acid
Source: BMC Plant Biol. 2023 Apr 6;23:185. doi: 10.1186/s12870-023-04161-7 (PMC10077631; doi:10.1186/s12870-023-04161-7)

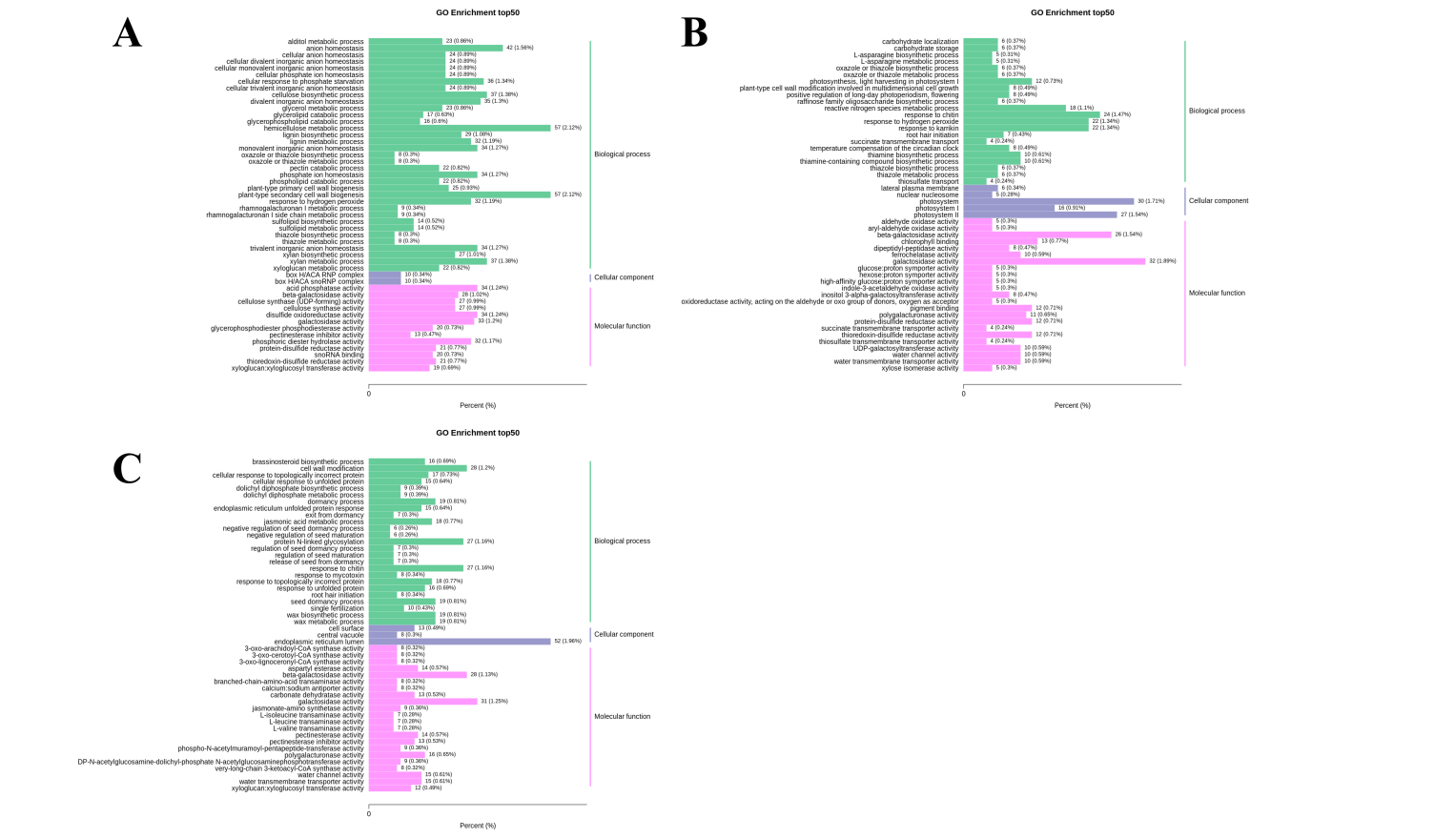


**Fig. S2** GO enrichment analysis. (A) T0 vs T1. (B) T0 vs T2. (C) T0 vs T4.

Supplement: Supplementary file 2 — Supplementary Material 2 [file 12870_2023_4161_MOESM2_ESM.docx]
